# Oxidative Status and Acute Phase Reactants in Patients with Environmental Asbestos Exposure and Mesothelioma

**DOI:** 10.1155/2014/902748

**Published:** 2014-01-23

**Authors:** Cengizhan Sezgi, Mahsuk Taylan, Hadice Selimoglu Sen, Osman Evliyaoğlu, Halide Kaya, Ozlem Abakay, Abdurrahman Abakay, Abdullah Cetin Tanrıkulu, Abdurrahman Senyiğit

**Affiliations:** ^1^Department of Pulmonary Diseases, School of Medicine, Dicle University, 21280 Diyarbakir, Turkey; ^2^Department of Biochemistry, School of Medicine, Dicle University, 21280 Diyarbakir, Turkey

## Abstract

*Background and Objectives*. The aim of this study was to investigate inflammatory indicators and oxidative status in patients with asbestos exposure with and without mesothelioma and to compare results with data from healthy subjects. *Methods*. Eighty people with exposure to environmental asbestos and without any disease, 46 mesothelioma patients, and a control group of 50 people without exposure to environmental asbestos were enrolled in this prospective study. Serum total oxidant level (TOL), total antioxidant capacity (TAC), and oxidative stress index (OSI), CRP, transferrin, ceruloplasmin, **α**-1 antitrypsin, ferritin, and copper levels were measured. *Results*. Mesothelioma group exhibited higher TOL, OSI, **α**1-antitrypsin, ferritin and copper levels as compared to the other groups (*P* < 0.001, *P* = 0.007, *P* < 0.0001, *P* < 0.001, and *P* < 0.001, resp.). Transferrin was lower in the mesothelioma group than in the other two groups (*P* < 0.001). The asbestos group had higher TOL, TAC, **α**1-antitrypsin, and transferrin levels (*P* < 0.001, *P* < 0.001, *P* < 0.001, and *P* < 0.001, resp.), as well as lower OSI and ferritin levels as compared to the control group (*P* < 0.001 and *P* < 0.001). *Conclusions*. We believe that elevated acute phase reactants and oxidative stress markers (TOL and OSI) in the mesothelioma group can be used as predictive markers for the development of asbestos-related malignancy.

## 1. Introduction

Asbestos causes pulmonary fibrosis, pleural diseases, and malignancies. However, the pathogenesis of asbestos-related diseases has not been clearly shown [1]. Recent studies indicate that increased production of reactive oxygen species (ROS) caused by asbestos plays an important role in this pathogenesis [2].

Oxidative stress occurs as a result of the failure to neutralize ROS with enzymatic and nonenzymatic systems [3]. Elevated levels of ROS lead to cell damage through peroxidation of double-chain fatty acids, protein, and DNA [4]. Moreover, ROS have been shown to cause apoptosis, inflammation, and proliferation [5]. Previous studies have shown that exposure to asbestos leads to oxidative stress by revealing reduced levels of antioxidant enzymes such as superoxide dismutase, catalase, glutathione peroxidase, and heme oxygenase [2, 6]. However, individual values of these enzymes may not correctly reflect total oxidant or total antioxidant status. Erel developed a new automated and colorimetric measurement method for total oxidant level (TOL) and total antioxidant capacity (TAC), while the ratio of these two parameters (TOL/TAC) is used for the calculation of oxidative stree index (OSI) which in turn allows the assessment of balance between ROS and antioxidant systems [7]. To our knowledge, there is not any study evaluating oxidative stress in malignant mesothelioma (MM) and asbestos exposure in such detail in the literature.

Chronic inflammation induced by different biologic and chemical factors has been shown to be a significant predisposing factor in the development of various organ cancers [8]. For example, chronic inflammatory bowel disease is a predisposing factor of colon cancer, chronic B and C hepatitis are predisposing factors of hepatocellular carcinoma, and chronic gastritis induced by Helicobacter pylori is a predisposing factor of gastric cancer [9–11]. Similarly, there are studies investigating the link between chronic inflammation associated with long-term asbestos exposure and mesothelioma [12, 13]. Authors claim that chronic inflammation triggered by asbestos exposure leads to increased production of ROS from inflammatory cells or alteration of immunocompetent cells and later reduction of tumor immunity [14, 15].

Acute phase reactants are plasma proteins that are synthesized by hepatocytes as a nonspesific response against tissue damage, infection, inflammation, trauma, or cancer. Acute phase reactants are frequently used in the evaluation of chronic inflammation in diseases such as inflammatory bowel disease or rheumatoid arthritis. Particularly C-reactive protein, fibrinogen, haptoglobin, ferritin, ceruloplasmin, copper, and *α*1-antitrypsin can be mentioned among the proteins that show notable increases during acute phase response. On the other hand, proteins such as albumin and transferrin demonstrate decreases during the response and are called as “negative acute phase reactants.”

Different diseases associated with asbestos may present with different levels of oxidative stress or inflammation. The importance of oxidative stress and inflammation can be assessed by various markers and modalities targeting this system can be established in the treatment and follow-up. In this study, we aim to evaluate oxidative markers including TOL, TAC, OSI, and inflammatory indicators and compare their relationship with each other in patients with asbestos exposure having no disease and in patients with asbestos exposure and MM.

## 2. Material and Methods

### 2.1. Study Subjects and Area

This cross-sectional study was conducted at the Pulmonology Department of Dicle University, Diyarbakir Southeastern Turkey. Environmental asbestos exposure is common in the southeast region of Turkey. In this region asbestos-containing soil is used to purpose of thermal insulation and waterproofing on roof and wall (material of whitewash-wall plaster). Environmental exposure occurs through inhalation of asbestos-laden soil.

Exposure with asbestos begins at birth in rural areas and the exposure is continuous. Thus patient's age was accepted exposure duration time in our study. We enrolled eighty villagers who have more than 20 years of environmental asbestos exposure were included in the study. Asbestos was detected in village in soil analysis. This village also was in an area that MM patients occure. There was not any finding for MM and other diseases in Chest X-ray in villagers. Forty-six patients with MM who were registered and followed up in our Department of Pulmonology were enrolled the study. The control group was created 50 healthy people with a mean of similar age and gender and who living in an area which not detected asbestos in soil analysis and without any disease.

The patients with chronic kidney failure, chronic heart failure, liver failure, and chronic obstructive pulmonary disease and those who have got active infection were excluded the study. The patients with malignancies other than MM were excluded from the study.

The study protocol was carried out in accordance with the Helsinki Declaration as revised in 1998 and approved by the local research committee for ethics. All subjects were informed about the study protocol and written consents were obtained from all inhabitants.

### 2.2. MM Diagnosis

Thoracentesis and closed pleural needle biopsy were performed in patients with pleural effusion for pathological and cytological examination. Ramel needle biopsy set was used in closed pleural biopsy. Surgical biopsy was performed when closed pleural biopsy is not appropriate The ultrasound-guided biopsy was performed in patients with small amount of pleural effusion. Tissue samples were immediately placed in 10% formol and sent for histopathological examination. Hematoxylin and eosin staining was used as standard in histopathological evaluation. Histochemical or immunohistochemical staining were used if necessary. The patients with confirmed MM diagnosis histopathologically were included in the study. Certain laboratory, clinical, and radiographic variables which measured at the time of diagnosis. were defined as potential prognostic factors.

### 2.3. Blood Sampling

To measure TOL and TAC, two 10 mL samples of blood were drawn from antecubital veins and were collected in empty tubes. Samples were separated from cells by centrifugation at 4000 rpm for 5 min, and then serum was stored at −80 C until analysis.

### 2.4. Measurement of TOL

TOL of serum was determined using an automated measurement method (Rel assay diagnostics kits, MegaTip, Gaziantep, Turkey) developed by Coussens and Werb [8]. Oxidants present in the sample oxidize the ferrous ion-o-dianisidine complex to ferric ion. The oxidation reaction is enhanced by glycerol molecules, which are abundantly present in the reaction medium. The ferric ion makes a colored complex with xylenol orange in an acidic medium. The color intensity, which can be measured spectrophotometrically, is related to the total amount of oxidant molecules present in the sample. The assay was cali-brated with hydrogen peroxide, and the results are expressed as micromoles of hydrogen peroxide equivalents per litre (mmol H_2_O_2_ equiv/L).

### 2.5. Measurement of TAC

Serum TAC levels were determined using a novel automated measurement method (Rel assay diagnostics kits, Mega Tip, Gaziantep, Turkey), developed by Coussens and Werb [8]. In this method, hydroxyl radical, which is the most potent radical, is produced via Fenton Reaction. In the classical Fenton reaction, the hydroxyl radical is produced by mixing ferrous ion solution and hydrogen peroxide solution. In the recently developed assay by Erel, the same reaction is used. In this assay, ferrous ion solution, which is present in Reagent 1, is mixed with hydrogen peroxide, which is present in Reagent 2. The sequentially produced radicals such as brown colored dianisidinyl radical cation, produced by the hydroxyl radical, are also potent radicals. In this assay, antioxidative effect of the sample against the potent-free radical reactions, which is initiated by the produced hydroxyl radical, is measured. The assay has got excellent precision values, which are lower than 3%. The results are expressed as mmol Trolox equiv./L.

### 2.6. Oxidative Stress Index (OSI)

The percent ratio of the TOL to the TAC gave the OSI, an indicator of the degree of oxidative stress. To perform the calculation, the result unit of TAC was changed to mmol Trolox equiv./L and the OSI value was calculated as below formula; OSI = [(TOL, mol/L)/(TAC, mmol Trolox equiv./L)*£*100.

C-reactive protein (CRP), transferrin, ceruloplasmin,  *α*-1 antitripsin, and ferritin levels were determined by immunonephelometric method with an autoanalyser (Image 800 Beckman Coulter, Fullerton, CA, USA); ve copper was determined by atomic absorption/emission spectrometer (Shimadzu 6401S Shimadzu Biotech, Kyoto, Japan).

### 2.7. Statistical Analysis

Data were stored in an Excel 2003 (Microsoft, Redmond, WA, USA) worksheet and loaded by statistics software SPSS 18.0 (SPSS Inc., Chicago, IL, USA) was used for statistical analysis. One way ANOVA was used to compare the values of the biochemical parameters of MM, asbestos exposure, and control groups. Differences in mean values of the parameters between subgroups were assessed for statistical significance by Tukey test. Results were presented as *n*, mean ± SD (standard deviation). Differences with a value of less than 0.05 were accepted as statistically significant.

## 3. Results

Mean age of MM group is 58.9 ± 12.3 years (23 females, 23 males), mean age asbestos group 61.7 ± 21.4 (44 women, 36 men) and mean age of control group 58.3 ± 16.2 (26 female, 24 male).

Table 1 summarizes serum TOL, TAC and OSI activities, CRP, transferrin, ceruloplasmin, *α*-1 antitrypsin, ferritin and copper profiles in MPM group, asbestos exposure group, and control.

As shown in Table 1, the mesothelioma group exhibited higher TOL (*P* < 0.001), OSI (*P* = 0.007), *α*1-antitrypsin (*P* < 0.001), ferritin (*P* < 0.001), and copper (*P* < 0.001) levels as compared to the other groups. The TAC level was lower in the mesothelioma group than in the asbestos group (*P* < 0.001), and it was similar in the mesothelioma and control groups (*P* = 0.074). Transferrin was lower in the mesothelioma group than in the other two groups (*P* < 0.001).

The asbestos group had higher TOL (*P* < 0.001), TAC (*P* < 0.001), *α*1-antitrypsin (*P* < 0.001), and transferrin (*P* < 0.001) levels, as well as lower OSI (*P* < 0.001) and ferritin (*P* < 0.001) levels as compared to the control group.

## 4. Discussion

TAC, TOL, and OSI have not been studied regarding their roles as oxidative stress markers in patients with environmental asbestos exposure. In our study, the asbestos and mesothelioma groups showed significantly higher TOL values, indicating that asbestos exposure leads to increased ROS levels. In the asbestos group, increasing TOL was observed to be balanced with increasing TAC, while oxidative stress was found to be at nonsignificant levels (low OSI). On the other hand, MM group showed higher increases in TOL levels as compared to the asbestos group, while also displaying inadequate balancing with antioxidants (low TAC) and a marked level of oxidative stress (high OSI).

Numerous papers suggest that oxidative stress caused by free radicals and ROS, plays a crucial role in the development of asbestos-induced lung disease [2, 15]. Asbestos leads to excessive ROS synthesis in two ways: (1) direct chemical catalyzer impact generated by the free iron ion content (Fe^+2^) (Fenton reaction) and (2) induction by the activation of inflammatory cells to the site of asbestos fiber deposition [2–4]. Stress occurs when the oxidative/antioxidative balance is shifted towards the oxidative side in tissues and organs. The oxidant burden results in multiple genetic alterations, such as facilitating mutations and/or inactivation of tumor suppression genes, and activating oncogenes [16]. In conclusion, oxidative stress can lead to cell injury by various pathways, or possibly, underbalanced conditions may initiate malignant transformation.

Previous in vitro cell culture and animal trials have shown that asbestos causes oxidative stress in the lung epithelium and pleural cells. Decreased levels of antioxidant enzymes such as superoxide dismutase, catalase, glutathione peroxidase, and heme oxygenase; and increased levels of oxidant markers such as MDA were reported [4–6, 17]. In the present study, elevated TOL in the asbestos and MM groups was a finding consistent with the previous studies [4–6, 17]. In the MM group, elevated TOL was observed to receive inadequate neutralization from TAC, leading to the development of a marked oxidative stress. Our study supports the idea that oxidative stress markers (TOL, TAC, and OSI) can be useful in the assessment of predisposition to MM development and early diagnosis among cases of asbestos exposure. Moreover, delivery of antioxidants in these individuals is believed to reduce the risk of MM development. However, further prospective studies including long-term follow-up of patients with asbestos exposure is needed.

Asbestos exposure is claimed to cause chronic inflammation, thus leading to alteration of immunocompetent cells and later reduction of tumor immunity [15].

High levels of CRP in the blood and pleural fluid and its correlation with survival has been reported in patients with MM [18–20]. In our study, CRP levels were comparable in the asbestos and control groups, indicating that inflammation was not remarkable in the asbestos group. However, CRP was significantly higher in the MM group than in the other groups. Elevated CRP level may be an indicator of development of asbestos-related diseases such as MM.

*α*1-antitrypsin is known to be active particularly in the protection of alveoli and liver, while also having a remarkable antioxidant property [21]. In one study, *α*1-antitrypsin level was associated with asbestos-related immunologic stimulation and diffuse pulmonary fibrosis [22]. Increased *α*1-antitrypsin level was found useful for diagnosis, metastasis, and recurrence evaluation in lung adenocarcinoma [23, 24]. In the present study, *α*1-antitrypsin was higher in the asbestos group than in the control group, whereas it was higher in the MM group than in the asbestos group. However, when we consider that *α*1-antitrypsin is both an inflammatory marker and an antioxidant, and that CRP level was comparable in the asbestos and control groups in our study, it is clear that the elevated *α*1-antitrypsin level in the asbestos group was a response against the TOS, not against the inflammation.

Transferrin is a negative acute phase reactant with a mild antioxidant property. In one study, serum transferrin level was found to be lower in the lung cancer group than in the control group and therefore it was noted as suitable for monitoring prognosis [25]. In our study, transferrin level was significantly higher in the asbestos group than in the control group, suggesting that the degree of inflammation was lower in the asbestos group. The transferrin level in the MM group was significantly lower as compared to the other two groups, indicating that the degree of inflammation in MM was more remarkable.

Ferritin is a protein in the body that binds to iron, while also acting as a positive acute phase reactant. In the present study, the ferritin level was significantly lower in the asbestos group than in the control group. Significantly elevated ferritin level in the MM group indicates a high-level inflammation. Studies have shown that serum ferritin is high in lung cancer patients and it has been noted as an important marker for the evaluation of performance status and prognosis [26]. In one study, the ferritin level was found to be higher in malignant pleural effusion than in benign pleural effusion [27].

An experimental study conducted in mesothelioma cases found raised copper levels in mesothelioma cells and reported copper as a possible marker of mesothelioma [28]. In our study, the copper levels were similar in the asbestos and control groups; however, MM group had a higher copper level as compared to the other two groups. Ceruloplasmin is a copper-carrying glycoprotein. It uses iron oxidase activity to prevent the occurrence of toxic iron products. In the present study, ceruloplasmin level was higher in the asbestos group than in the control group, while it was also higher in the MM group than in the asbestos group. One study investigated acute phase reactants in rats with asbestos exposure and found elevated ceruloplasmin concentrations [29]. In another study, ceruloplasmin levels were observed to be high in lung cancer [30]. In a study in Finland, high serum ceruloplasmin concentrations during the years prior to diagnosis were associated with an increased risk of cancer, especially with lung cancer [31].

Determining markers that can predict MM development following environmental asbestos exposure are an important research field. Further prospective studies including long follow-up periods are needed to more clearly understand the predictive efficacy of inflammatory and oxidative markers.

In conclusion, the presence of higher transferrin, lower ferritin, and similar CRP levels in the asbestos group as compared to the control group shows the inflammation in the background. However, we believe that elevated acute phase reactants and oxidative stress markers (TOL and OSI) in the MM group can be used as predictive markers for the development of asbestos-related malignancy.

## Figures and Tables

**Table 1:** The profiles of markers in different groups.

	Control	Asbestos	Mesothelioma	*P*
TOL (mmol H_2_O_2_ equiv./L)	105.9 ± 92.5	145.1 ± 71.9^b^	196.3 ± 221.1^c,d^	0.001
TAC (mmol Trolox equiv./L)	0.73 ± 0.34	1.27 ± 0.16^c^	0.8 ± 0.3^f^	<0.001
OSI	159.3 ± 160.9	112.8 ± 48.3^c^	898.6 ± 129.7^b,f^	0.007
CRP (mg/dL)	2.8 ± 1.7	4.4 ± 3.1	75.6 ± 54.8^a,f^	<0.001
*α*-1 antitrypsin (mg/dL)	146.7 ± 47.3	213.9 ± 33.9^c^	262.4 ± 94.9^c,f^	<0.001
Transferrin (g/L)	216.9 ± 62.3	282.8 ± 50.1^c^	171.2 ± 44.5^c,f^	<0.001
Ferritin (ng/mL)	112.3 ± 43.7	85.4 ± 110.6	370.7 ± 267.8^c,f^	<0.001
Copper (*μ*g/mL)	0.8 ± 0.2	0.7 ± 0.3	1.2 ± 0.3^c,f^	<0.001
Ceruloplasmin (mg/L)	36.6 ± 8.6	40.1 ± 9.7	42.9 ± 8.2^c,d^	0.001

TOL: total oxidant level; TAC: total antioxidant capacity; OSI: oxidative stree index; CRP: C-reactive protein; ^a^significantly different from control (*P* < 0.05), ^b^significantly different from control (*P* < 0.01), ^c^significantly different from control (*P* < 0.001), ^d^significantly different from asbestos (*P* < 0.05), ^e^significantly different from asbestos (*P* < 0.01), and ^f^significantly different from asbestos (*P* < 0.001).
